# Broad-Host-Range Synthetic Biology: Rethinking Microbial Chassis as a Design Variable

**DOI:** 10.1021/acssynbio.5c00308

**Published:** 2025-09-18

**Authors:** Dennis Tin Chat Chan, Johan Bjerg, Hans C. Bernstein

**Affiliations:** 1 Faculty of Biosciences, Fisheries and Economics, UiT - The Arctic University of Norway, Tromsø 9019, Norway; 2 Biochemical Engineering Group, Plant Biochemistry Section, Department of Plant and Environmental Sciences, University of Copenhagen, Thorvaldsensvej 40, 1871, Frederiksberg C, Denmark; 3 Department of Biotechnology and Biomedicine, Technical University of Denmark, 2800 Kongens Lyngby, Denmark

## Abstract

Broad-host-range synthetic microbiology is redefining the role of microbial hosts in genetic design by moving beyond the traditional organisms. Historically, synthetic biology has focused on optimizing engineered genetic constructs within a limited set of well-characterized chassis, often treating host-context dependency as an obstacle. However, emerging research demonstrates that host selection is a crucial design parameter that influences the behavior of engineered genetic devices through resource allocation, metabolic interactions, and regulatory crosstalk. By leveraging microbial diversity, broad-host-range synthetic biology enhances the functional versatility of engineered biological systems, enabling a larger design space for biotechnology applications in biomanufacturing, environmental remediation, and therapeutics. The continued development of broad-host-range tools—including modular vectors and host-agnostic genetic devices—facilitates the expansion of chassis selection, improving system predictability and stability. This perspective highlights the advantages of incorporating host selection into synthetic biology design principles, positioning microbial chassis as tunable components rather than passive platforms.

## Graphical abstract

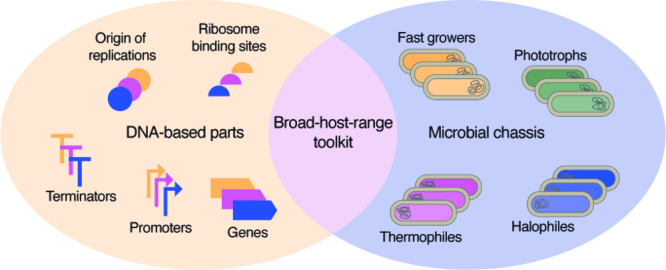

## 1 Introduction

Broad-host-range (BHR) synthetic biology has emerged as a modern subdiscipline of bioengineering that focuses on the use of nontraditional organisms as host platforms with the goal of expanding current biodesign capabilities. Historically, synthetic biology has been biased toward using a narrow set of traditional organisms (e.g., *Escherichia coli* and *Saccharomyces cerevisiae*) as chassis due to their genetic tractability and the availability of robust engineering toolkits.^1^ While these workhorse organisms have been invaluable for demonstrating proof-of-concept systems, they might not represent the most optimal chassis for a given application.^2^ There likely exist other organisms in nature capable of outperforming a traditional organism as a chassis for any given bioengineering goal.^3,4^ The bias toward traditional organisms can thereby be viewed as a design constraint self-imposed by synthetic biologists that has consequently left the chassis-design space an untapped area of engineering potential. BHR synthetic biology aims to alleviate this constraint by promoting the exploration of the chassis-design space and the use of nontraditional hosts as chassis. Another aim is the reconceptualization of the chassis as an integral design variable that should be rationally chosen with the goal of optimizing system function rather than a parameter that is defaulted to a traditional organism.

The emergence of BHR synthetic biology is deeply rooted in the history of synthetic biology as a discipline focused on applying engineering principles to biological systems.^5^ The term “broad-host-range” has historically referred to DNA parts such as promoters, terminators, and origin of replication sequences, which dates back to 1995 ^6^ but has more recently also been used to refer to engineered genetic devices^7–11^ and plasmid vectors^12,13^ that function across multiple host organisms such as the Standard European Vector Architecture (SEVA).^14^ Since its inception over two decades ago, synthetic biology has prioritized abstraction, modularity, and the design-build-test-learn cycle to program cellular behavior.^15^ These tools and frameworks have broadened the field of synthetic microbiology but also highlighted a key limitation: reliance on a narrow range of well-characterized organisms.^16^ As synthetic biology progresses, the need to move beyond traditional chassis and explore a wider diversity of microbial hosts to enhance functional capabilities becomes a necessary area of focus.^17–20^ However, it has remained a significant challenge to deploy the same advanced biodesign principles in nontraditional hosts.^21^ Despite the increasing number of domesticated microbial hosts available for biotechnology, synthetic biologists face significant challenges when venturing beyond the established traditional organisms.^22^ One key challenge is the entrenched assumption that the host organism primarily serves as a passive provider of resources and machinery,^23,24^ leaving the optimization of genetic device performance to be done almost exclusively within the genetic context (*e.g.,* circuit architecture and parts selection). Furthermore, there is a lack of research on how engineered genetic constructs perform across diverse host contexts,^7–9^ which hinders accurate cross-species predictions and, in turn, discourages exploration beyond traditional organisms. In this Perspective, we highlight recent advances and outline an emerging scope of opportunity that will help overcome these two barriers and progress the field of microbial synthetic biology.

## 2 Reconceptualizing the Role of Microbial Hosts in Genetic Design

By reframing host selection as a functional parameter, synthetic biologists can take advantage of host-specific traits to construct new functions or improve native functions. Contemporary biodesign involves introducing some form of genetic machinery (such as a circuit) into a host organism to confer the host with augmented functionality.^25^ In the traditional and most widely adopted approach, novel functions (e.g., biosynthesis of medicinal compounds from sustainable substrates) and their subsequent tuning are done through exploration of the engineered genetic components such as promoters, RBS, coding sequences, codon optimizations, etc. (Figure 1a) while the chassis defaults to a “model” organism. This approach dominated the early days of synthetic biology and led to early foundational breakthroughs, such as the construction of the genetic toggle switch^26^ and synthetic oscillator^27^ in *E. coli*. Contrary to the traditional approach, BHR efforts encourage the exploration of the host context. A core principle of BHR synthetic biology is that the host chassis should be treated as a modular part (Figure 1b). For this purpose, the chassis can serve as a “functional” module and/or as a “tuning” module. As a functional module, the innate traits of the chassis are integrated into the design, often serving as the foundation from which the design concept originates. For example, the native photosynthetic capabilities of phototrophs (e.g., cyanobacteria^28,29^ and some genetically tractable microalgae^30,31^ ) can be rewired for the biosynthetic production of value-added compounds from carbon dioxide and sunlight. Similarly, many organisms have been specifically developed as synthetic biology chassis due to their natural ability to produce value-added compounds, such as fucoxanthin^32^ and terpenoids^33^. Furthermore, the natural tolerance of thermophiles,^34,35^ psychrophiles,^36^ and halophiles^37–39^ makes them well-suited as chassis for development of biosensors, bioremediation agents, large-scale fermenters, or any process requiring robust performance in harsh nonlaboratory environments.

**Figure 1:**
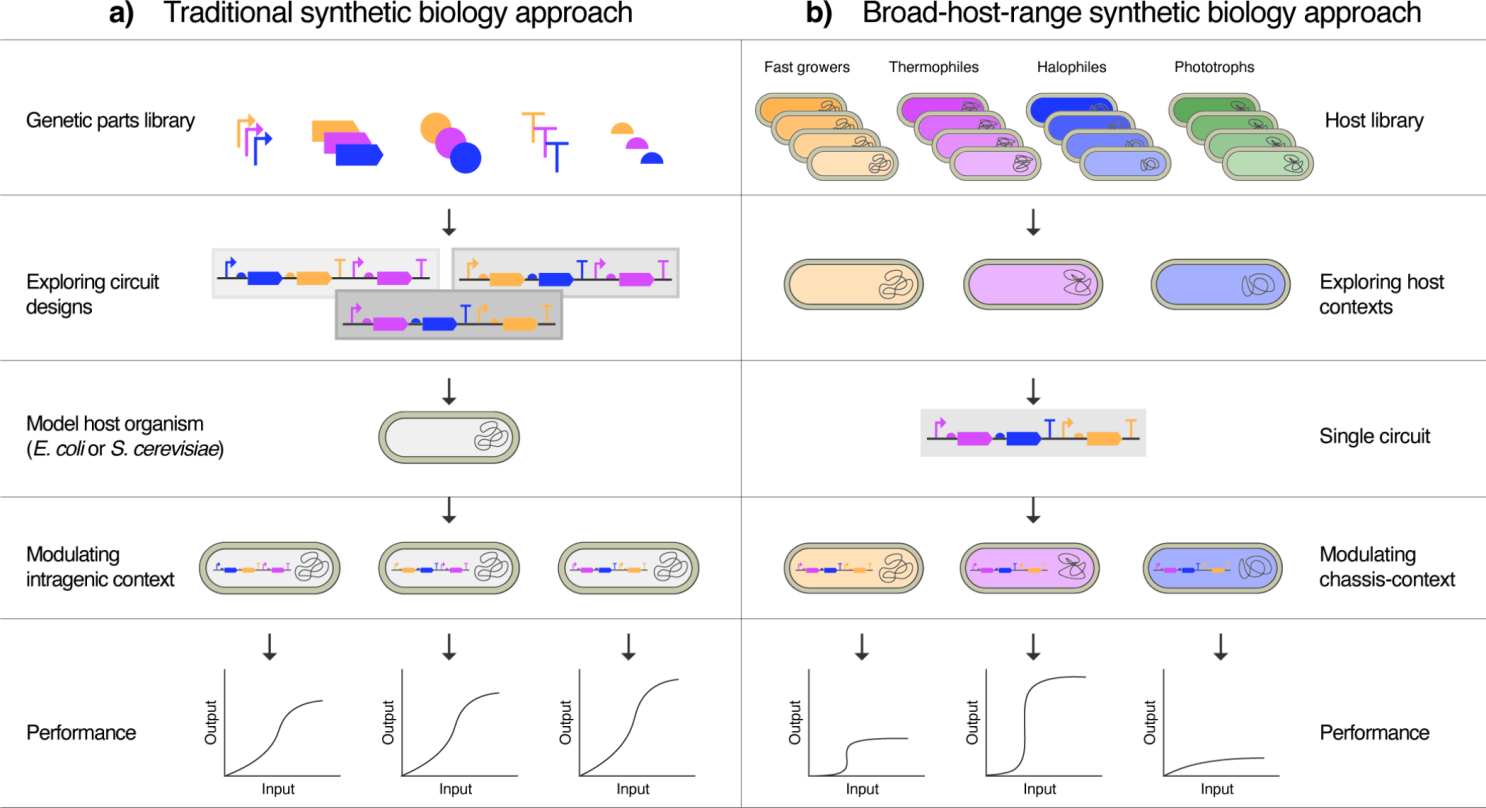
Conceptual comparison between traditional and host-centric approaches in synthetic biology. Traditional biodesign workflows focus on tuning genetic device components—such as promoters, RBSs, and coding sequences—within a limited set of traditional hosts, treating the chassis as a passive background. In contrast, broad-host-range (BHR) synthetic biology reframes the chassis as an active and tunable component of the design process. This host-centric approach integrates microbial diversity into biodesign, enabling synthetic biologists to leverage host-specific traits to optimize system performance. The figure illustrates how BHR strategies expand the design space by coupling genetic device design with strategic chassis selection, offering a complementary route to achieving desired functions.

Examples of organisms that were specifically domesticated for their pragmatic phenotypes include the metabolically versatile *Rhodopseudomonas palustris* CGA009, a purple nonsulfur bacterium capable of all four modes of metabolism^17^ with potential as a growth-robust chassis, as well as a number of members of the *Halomonas* genus,^40^ notably *Halomonas bluephagenesis*,^41,42^ for their high-salinity tolerance and natural product accumulation. Besides prokaryotic microbes, synthetic biology has been applied to optimize the catalytic and biomanufacturing capabilities of filamentous fungi^43^ and diatoms^44^ (e.g., *Phaeodactylum tricornutum* ^45^). Having a biologically diverse set of chassis available gives options for a user to select the most optimal “host-canvas” for a specific design goal. Retrofitting the preengineered phenotypes of an organism into artifical designs is arguably more cost-beneficial than attempting to engineer forth the same phenotype (e.g., a biosynthetic pathway, photosynthesis, or high-temperature tolerance) in a traditional organism. This concept of “hijacking” nature is not new in the field of synthetic biology. Early synthetic biologists already recognized the limitations of *E. coli* as a chassis, for instance, when the expression of eukaryotic genes is desired. The correct expression of certain human genes, such as G-protein coupled receptors (GPCRs), requires an environment permissive for the correct folding of the receptor as well as the necessary post-translational modifications and trafficking.^46^ Expressing functional and properly membrane-localized GPCRs is therefore a great challenge in bacteria, which lack the processing organelles and molecular machinery needed. Yeast, however, already harbors a native GPCR signaling pathway that can be modularized to establish human GPCR biosensors that can be used for drug target exploration.^44^ It should be noted that our view of BHR synthetic biology does not aim to replace the traditional approach but rather to expand the current design space by coupling biodesign with strategic chassis selection, offering a complementary route to achieving desired functions.

Besides acting as a functional module, the chassis can be used as a tuning module to adjust the performance of genetic circuits. As a tuning module, the function of the circuit is often independent of any host phenotype, but the circuit performance specifications are influenced by the host environment. Recent studies have demonstrated how identical genetic circuits, such as inverting switches, can exhibit different performance metrics when operating within the unique cellular environments of different hosts, revealing novel ways to optimize responsiveness, sensitivity, and stability^9,10,47^ (Figure 2). Systematic comparisons of genetic circuit behavior across multiple bacterial species have shown that host selection can significantly influence key parameters such as output signal strength, response time, growth burden, and expression of native carbon and energy pathways,^7,8^ providing a spectrum of performance profiles that synthetic biologists can leverage when choosing a functional system.

**Figure 2:**
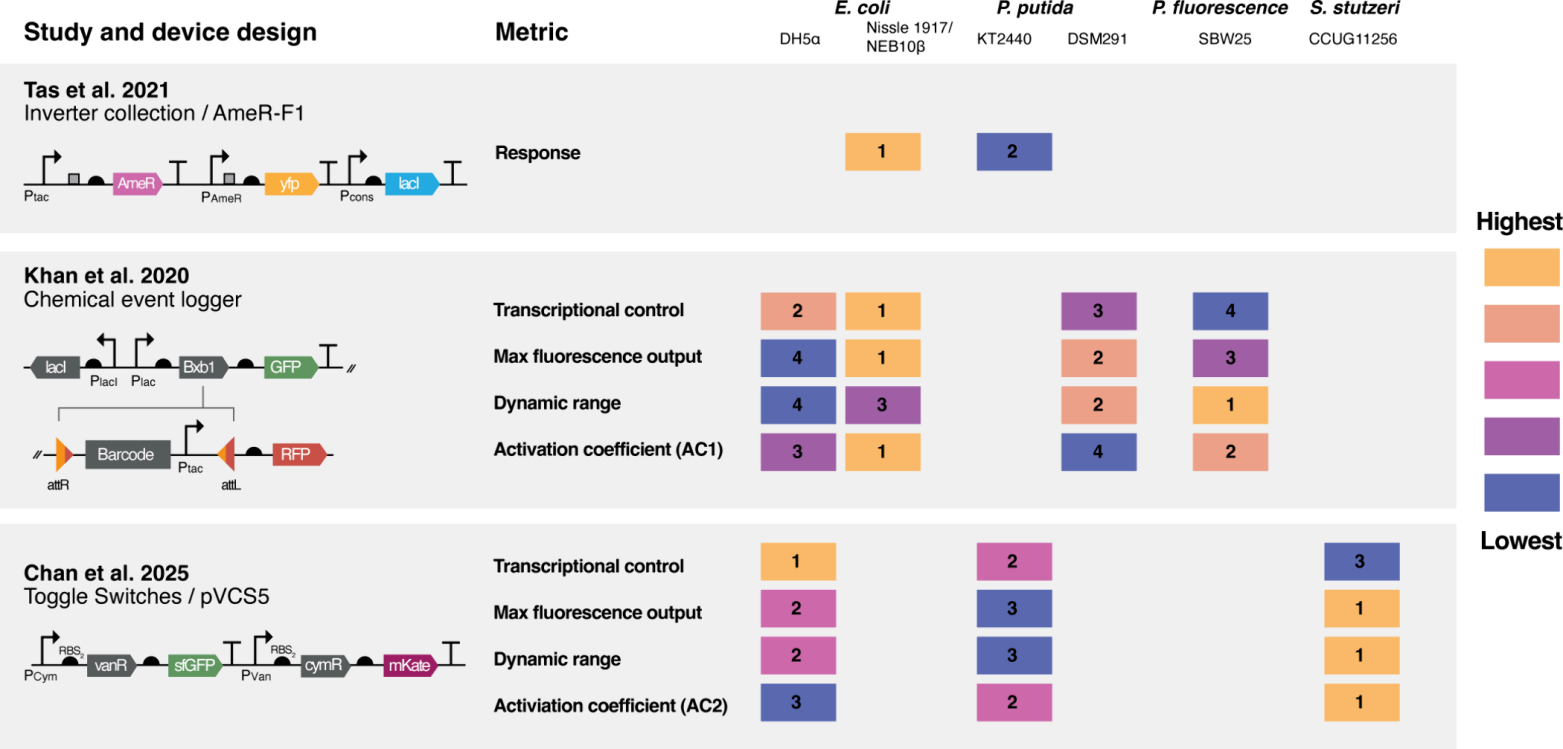
Comparative summary of genetic device performance across*E. coli* strains and alternative bacterial hosts highlights the chassis effect. This figure integrates data from three studies that evaluated engineered genetic constructs in different bacterial strains, illustrating how device performance varies as a function of host context. Strain and device combinations are ranked from lowest to highest for each reported performance metric within each study, showcasing distinct functional profiles shaped by the chassis. From Tas et al.,^47^ the inverter device AmeR-F1 was selected from a library of 20 designs and tested in *E. coli* NEB10β alongside environmental isolates. From Khan et al.,^13^ a chemical event logger based on a Bxb1 integrase was assessed in *E. coli* DH5α and Nissle 1917 and two *Pseudomonas* species, with the recombination sites (*attR* and *attL*) shown here in their inverted state. The study from Chan et al.^8,9^ included several of the same comparative strains but also included *Stutzerimonas stutzeri* CCUG11256 engineered with pVCS5 constructed from 9 variants of a vanillate/cumate inducible toggle switch, with performance ranked based on output intensity and response parameters. Design elements are indicated by genetic glyphs: perpendicular arrow (promoter), squares (ribozyme site), half-circle (ribosome binding site), directional box (coding sequence), T (terminator), and two-colored triangles (recombination sites). Additional metrics include AC1 (half-saturation time for GFP and RFP expression) and AC2 (inducer concentration for half-maximal output). Together, these cross-study comparisons demonstrate the variability introduced by host-circuit interactions, known as the chassis effect, and reinforce the need to treat host selection as a critical parameter in synthetic biology design.

Prioritizing chassis traits such as high transformation efficiency, high burden tolerance, robust growth across conditions, and compatibility with regulatory elements (e.g., sigma factors and transcriptional machinery) can improve construct predictability. However, host selection often involves trade-offs, for example, between sensitivity and total output, which are influenced by how a chassis allocates its internal resources. Therefore, the optimal host depends on application-specific goals, including not just device performance but also the ecological, metabolic, and operational contexts in which the chassis must function. Readers seeking further technical discussion of chassis-dependent device performance, physiological predictors, and host-circuit modeling are referred to recent works by Chan et al.,^7–9^ Khan et al.,^13^ and Tas et al.^11,47^

## 3 Chassis Effect and Other Roadblocks

The ability of synthetic biologists to precisely engineer exogenous genetic constructs down to the single-nucleotide level has enabled remarkable advancements in biodesign.^48^ However, predicting expression behavior *in vivo* remains a major challenge due to host-construct interactions and the inherent context dependency of operational genetic devices.^18,49^ The “chassis effect” refers to this phenomenon in which the same genetic manipulation exhibits different behaviors depending on the host organism it is operating within.^7^ The expression of exogenous gene products perturbs the host’s metabolic state, triggering resource reallocation that can influence function and lead to unintended changes in performance.^50^ Prior studies have demonstrated that resource competition^51,52^ and growth feedback^53,54^ shape genetic circuit behavior in unpredictable ways. For example, Espah Borujeni et al. showed how RNA polymerase flux and ribosome occupancy impact circuit dynamics,^55^ while Gyorgy modeled resource-competition effects on performance.^51^ Other specific mechanisms include divergence in promoter–sigma factor interactions,^51^ differences in transcription factor structure or abundance,^8^ and temperature-dependent RNA folding,^56^ all of which modulate gene expression profiles across hosts. These interactions arise from the coupling of endogenous cellular activity with the introduced genetic circuitry, either through direct molecular interactions (*e.g.,* transcription factor crosstalk and sequestration) or through competition for finite cellular resources such as ribosomes, RNA polymerase, and metabolites.^57,58^ Often, these host-circuit interactions lead to nonviable systems where the growth burden is too taxing on the host or leads to selection of systems with mutations debilitating to circuit function.^49^ The chassis effect can thereby represent an obstacle that prevents an accurate prediction of the circuit performance across hosts. The complex interplay between host metabolism and genetic circuitry makes it difficult to predict circuit performance solely on the basis of DNA sequence, discouraging the exploration of the chassis-design space. In a recent comparative study across *Stutzerimonas* species,^8^ the same inducible toggle switch circuit exhibited divergent bistability, leakiness, and response time—correlated with variation in host-specific gene expression patterns from their shared core genome, demonstrating how subtle genetic and physiological host differences can significantly alter device behavior. Another comparative study found differences in bacterial physiology to be the main determinant of differences in the performance of a genetic inverter device,^7^ with phylogeny being a poor determinant, the latter finding corroborating previous cross-species studies.^59,60^ While these studies show that the chassis effect can be traced to measurable differences in cellular states, the field is still far from developing a cross-host predictive model, necessitating a deeper investigation into cross-host performances and host-specific factors.

As a result of the chassis effect, numerous cases have been reported (with likely even more nonreported) where engineered circuits, although correctly assembled, fail to function as intended due to unanticipated host effects, excessive growth burden, toxic gene products, or immune responses.^53,57,61^ Even when circuits do function, performance characteristics such as response time, dynamic range, and output levels can vary significantly between hosts.^16,49,62^ However, rather than viewing these variations as confounding factors, BHR synthetic biology proposes that host selection should be deliberately leveraged as a part of circuit optimization strategies. This shifting viewpoint on the role of the host chassis does not reject orthogonalization^63,64^ or genome reduction strategies,^65,66^ but instead suggests a complementary approach, both in practice and in concept, where host diversity and targeted modifications can be used in tandem to improve system stability and efficiency.^67–69^ Selective genome reduction has been used to enhance growth and metabolic output in traditional strains of *E. coli*, *Bacillus subtilis*, and *Pseudomonas putida* ^70–72^ and can equally be applied to nontraditional hosts with established genetic tractability. This, however, would require the development of universal and portable engineering techniques. Future developments in BHR synthetic biology will integrate these strategies, leading to more adaptable and application-specific microbial systems.

Another major roadblock halting more widespread adoption of the BHR approach is that domestication of new hosts is a laborious and time-consuming process. For an organism to be considered domesticated, it must have a sizable genetic toolbox available (enabling selection and tuning of gene expression), an efficient transformation protocol, and cultivation methods.^73^ Establishing a genetic toolbox is usually the most time-consuming effort, but the availability of public repositories such as SEVA,^74^ AddGene,^75^ and SynBioHub^76^ has allowed researchers to screen through standardized libraries of tools that can or have already been adapted for BHR. This is further empowered by scalable and standardized DNA assembly techniques and automation. Still, adapting existing methods such as CRISPR^77^ or CRISPR Optimized MAGE Recombineering^78^ for genomic modifications to a novel organism often requires extensive optimization to reach efficient levels. Note that previous bottlenecks, such as genome sequencing of cultivable isolates, have become practically obsolete given the democratization of sequencing technology. Further advancements in automation in terms of cost and power will likely eliminate synthetic biology bottlenecks such as cloning and sample screening.

As synthetic biologists seek to leverage host diversity, selecting an appropriate chassis requires a consideration of specific biological and operational parameters. These include transformation efficiency, growth rate, native stress tolerance, regulatory compatibility (e.g., sigma factor and TF divergence), and metabolic load tolerance. Furthermore, practical considerations such as genetic tool availability, safety classification, and cultivation cost must be factored into the design space. Explicitly defining these priorities during chassis selection will help to align host capabilities with circuit design goals.

## 4 Conclusions

BHR synthetic microbiology represents a necessary evolution in synthetic biology to move beyond traditional organisms to expand current biodesign capabilities. This shift is not just about expanding the chassis repertoire but also rethinking how host-context influences the performance and predictability of engineered biological systems. By integrating microbial diversity into synthetic biology workflows, researchers gain access to novel cellular environments that can optimize function, enhance stability, and unlock new biochemical capabilities that traditional chassis cannot provide.

To advance a future host-specific design framework, several concrete steps are needed. First, synthetic biologists must systematically characterize host-specific physiological and regulatory features—such as growth-coupled gene expression, metabolic resource allocation, genome structure, and stress tolerance—to inform predictive design. Recent studies have used multivariate models to correlate host features (e.g., genome-relatedness, physiological metrics, and transcriptomic profiles) with circuit performance.^4–6^ Building on this, curated databases should be developed that combine standardized genetic device performance data with host genome sequences, multiomics profiles, and experimentally validated phenotypes. Integrating these data streams into iterative design-build-test-learn loops would enable the required databases and training data for future predictive modeling of chassis effects and support informed host selection. Existing genome and omics databases (e.g., NCBI, ENA, IMG, and JGI) can serve as foundational layers. Furthermore, expanding metadata reporting standards in synthetic biology parts registries—such as linking parts to chassis compatibility across taxonomic lineages and host phenotypes—will allow rational extrapolation to related species. Ultimately, these tools will guide users toward selecting context-appropriate chassis for a given design or application.

The continued focus on BHR synthetic microbiology is not only enabling more robust and versatile biotechnologies but also deepening our understanding of fundamental biological mechanisms. Studying genetic constructs across diverse hosts reveals insights into resource allocation, cellular stress responses, and evolutionary constraints that would otherwise be obscured in a single model system. As synthetic biology advances toward more complex and application-driven designs, the strategic selection and engineering of nontraditional hosts will be key to addressing global challenges in sustainable manufacturing, environmental sustainability, and therapeutic development. By embracing the chassis as a tunable design variable, BHR synthetic biology provides a powerful framework for innovation, ensuring that synthetic biology remains adaptable and impactful across diverse biotechnological landscapes.
